# Rethinking the Ecosystem Functions of *Dicranopteris*, a Widespread Genus of Ferns

**DOI:** 10.3389/fpls.2020.581513

**Published:** 2021-01-13

**Authors:** Long Yang, Yuhui Huang, Lucas Vieira Lima, Zhongyu Sun, Meijie Liu, Jun Wang, Nan Liu, Hai Ren

**Affiliations:** ^1^Guangdong Open Laboratory of Geospatial Information Technology and Application, Guangdong Academy of Sciences, Guangzhou Institute of Geography, Guangzhou, China; ^2^Guangdong Provincial Key Laboratory of Forest Silviculture, Protection and Utilization, Guangdong Academy of Forestry, Guangzhou, China; ^3^Departamento de Botânica, Laboratório de Sistemática Vegetal, Instituto de Ciências Biológicas, Universidade Federal de Minas Gerais, Belo Horizonte, Brazil; ^4^Key Laboratory of Vegetation Restoration and Management of Degraded Ecosystems, South China Botanical Garden, Chinese Academy of Sciences, Guangzhou, China

**Keywords:** Dicranopteris, ecosystem function, facilitation and competition, ecosystem resilience, succession facilitation, tropical forest

## Abstract

*Dicranopteris* is an ancient and widespread genus of ferns in pantropical regions. Some species of the genus can form dense thickets, and dominate the understory, which are common and key species in tropical and subtropical ecosystems. However, they were mostly cut or burned in forest management because of forming dense thickets which were considered to interfere with forest regeneration and succession. In the current review, we argue that the *Dicranopteris* species which are able to rapidly colonize barren areas may contribute to ecosystem recovery, resistance to environmental stress, and succession control. Rapid colonization involves prolific spore production, rapid clonal growth, the generation of high surface cover, and the ability to fill gaps; stress resistance includes resistance to abiotic stress, and the ability to reduce soil erosion from rainfall, alien species invasion, and soil contamination and toxicity; and succession facilitation consists of carbon and nutrient sequestration in soil, moderation of the microclimate, alteration of the soil microbial and faunal communities, and determination of which plant species to be established in the next successional stage. All of these ecosystem functions may be beneficial to ecosystem resilience. We expect that the distribution of *Dicranopteris* will expand in response to global warming, changes in precipitation patterns, increases in soil pollution, deforestation, and land degradation. We recommend that *Dicranopteris*, as a pioneer fern and a valuable component of tropical and subtropical ecosystems, needs more attention in future research and better management practices to promote forest regeneration and succession.

## Introduction

**Figure 1:**
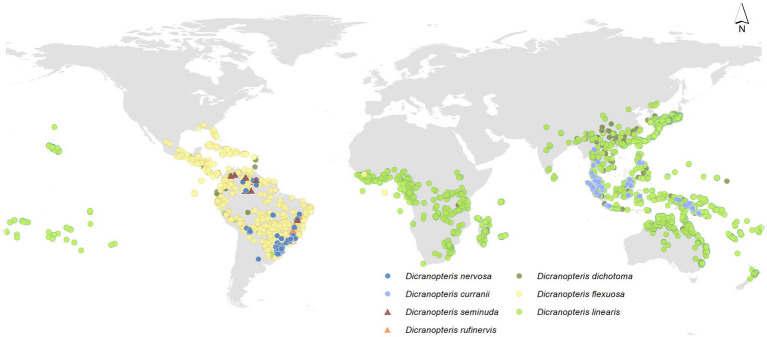
Global distribution of *Dicranopteris* species, including *Dicranopteris dichotoma* (deep green dots), *Dicranopteris curranii* (shallow blue dots), *Dicranopteris flexuosa* (yellow dots), *Dicranopteris seminuda* (brown triangles), *Dicranopteris nervosa* (navy blue dots), *Dicranopteris rufinervis* (orange triangles), and *Dicranopteris linearis* (green dots). The distribution information was mainly obtained from Global Biodiversity Information Facility (gbif.org), sampling by Long Yang in East Asia and Lucas Vieira Lima in the neotropics.

*Dicranopteris* (forked fern) is a genus of about 12 extant, common pioneer species in pantropical regions (Pteridophyte Phylogeny Group, 2016), which is an ancient fern genus of the family Gleicheniaceae (Filicopsida). The origin of ferns is estimated to have occurred in the mid-Silurian (Testo and Sundue, 2016). It is estimated that *Dicranopteris* diverged from Gleichenella about 38 m.a., which places the rise of the genus in late Eocene (Liu et al., 2020). Among them, six *Dicranopteris* species can form dense thicket (Figure 1). Some *Dicranopteris* species are concentrated in Asia, such as *Dicranopteris dichotoma* in East Asia and *Dicranopteris curranii* in Southeast Asia. In the neotropical region, there are four species of *Dicranopteris* of which *Dicranopteris flexuosa* is the most common and widespread species. The other *Dicranopteris* species include *Dicranopteris seminuda* restricted to the Andes, *Dicranopteris nervosa* with populations in south and southeastern Brazil, Bolivia, and Peru, and *Dicranopteris rufinervis* endemic to Brazil (Lima and Salino, 2018).

Plants of *Dicranopteris* consist of fronds, aerial stems, rhizomes, and adventitious roots. Pinnae and aerial stems constitute the aboveground part, whereas the underground stems and adventitious roots constitute the underground part (Liu et al., 2008). *Dicranopteris* are scrambling plants with pseudodichotomously branched fronds, which can reach 45–200 cm in height (Zhang et al., 2010). The rhizome may grow buried in soil or close to surface. The rhizome is long-creeping, and the fronds may reach up to 2 m in length. In most cases, the fronds are pendant and the new fronds rest on the older ones, usually forming dense thickets (Andersen and Øllgaard, 1996; Mickel and Smith, 2004; Lima and Salino, 2018). The aboveground part can intercept and retain wind-dispersed seeds by reducing wind velocity and increase the coverage and retention of animal-dispersed seeds as well. The underground part of the rhizomes and adventitious roots are arranged in a crisscrossed pattern, which resembles a fine sieve that is filled with litter and soil. Tropical and subtropical regions have received considerable attention from ecologists because they have huge carbon (C) pools (Phillips et al., 1998; Mitchard, 2018) and high biodiversity (Molino and Sabatier, 2001; Volkov et al., 2005), as well as provide many ecosystem services (Hietz et al., 2011; Hirota et al., 2011). Previous studies of ecosystem functions in tropical and subtropical regions primarily focus on dominant and overstory species of gymnosperms and angiosperms rather than on pioneer fern species (Molino and Sabatier, 2001; Volkov et al., 2005). Although such pioneer fern species dominate in the early stages of forest succession (Walker, 1994; Walker and Boneta, 1995; Walker and Sharpe, 2010), their ecological functions in general and contributions to succession in particular have rarely been studied due to their low biomass and diversity (Wu et al., 2011; Yang et al., 2014). *Dicranopteris* species have often been considered as competitive obstacles to forest regeneration and succession because of thickets that they form (Huan et al., 1984; Cohen et al., 1995; Slocum et al., 2004, 2006; Pang et al., 2018). In contrast, after reviewing recent studies, we found that *Dicranopteris* species may have important ecosystem functions. Especially Walker and Sharpe (2010) already covered both effects of Gleicheniaceae, inhibition and facilitation. Finally, we performed a comparison of the ecosystem functions between *Dicranopteris* and another widespread and often invasive genus, *Pteridium*, which has been studied far better than the former genus. After describing how *Dicranopteris* species may help support succession in pantropical ecosystems, we suggest future research directions in the context of global environmental change.

## Past Understanding of the Ecosystem Functions of *Dicranopteris*

*Dicranopteris* species generally form a single-species dominant community due to their superior propagation ability (Lin, 2004) and allelopathic effects (Yuan and Li, 2007). First, they can propagate through sexual reproduction (spores) and vegetative reproduction (clones) to form single-species dominant community. Vegetative propagation is more important for fast growth and occupying ecological space. Second, the allelopathic effect leads to difficult recruitment of other species. During the early successional stage of tropical and subtropical plant communities, *Dicranopteris* species are common and keystone taxa. However, the role of *Dicranopteris* is controversial in tropical ecology. *Dicranopteris* species have often been considered as obstacles that interfere with forest succession because they form thickets which compete with trees for soil water, soil nutrients, and solar radiation. This assessment has been applied to *D. dichotoma* in South China (Huan et al., 1984; Pang et al., 2018) and *Dicranopteris linearis* in Sri Lanka (Cohen et al., 1995) and Hawaii (Russell, 1996). In the short run, *Dicranopteris* species can inhibit seedling regeneration of late-successional species. However, in the long term, ecosystems are able to undergo succession (Chua et al., 2013).

In degraded land of South China, the aboveground biomass and rhizomes of *D. linearis* have often been burnt to slow down their growth and regeneration, the degraded land was turned over so that the rhizomes and roots were exposed to the sun, and then legumes were planted to replace the *Dicranopteris* (Huan et al., 1984). In montane forest in the Dominican Republic, woody species of different successional stages were planted to increase plant diversity after clearing the *Dicranopteris* thickets (Slocum et al., 2006). Bracken fern (*Pteridium*) has similar situation with *Dicranopteris*. *Pteridium* was described as an aggressive pioneer plant species worldwide (Le Duc et al., 2007). *Pteridium* can also form dense thickets for stronger allelopathic chemicals and rapid clone propagation. Thus, many control measures were carried out to restore the primary vegetation. Compared with the treatment of cutting once per year or asulam spraying, cutting twice per year can effectively control the expansion of invasive weed *Pteridium* in Great Britain (Cox et al., 2007; Le Duc et al., 2007; Stewart et al., 2008; Milligan et al., 2018).

*Dicranopteris* species share the following characteristics at the local scale: dense growth, the ability to form near monocultures, and a deep root blanket. All *Dicranopteris* species require strong sunlight, and commonly grow in pioneer communities, on barren slopes, or in sparse forests (Zhu et al., 2016). As the most common pioneer species in tropical and subtropical regions, *Dicranopteris* species play an essential role in microclimate formation, nutrient accumulation, nutrient cycling, and energy flux (Duan et al., 2010). Compared to ferns that grow in more shaded environments, *Dicranopteris* species have higher nutrient-use efficiencies and shorter leaf life spans and payback times (Zhu et al., 2016). However, the ecosystem functions of *Dicranopteris* have not been well acknowledged in previous studies. In the following sections, we summarize the ecosystem functions of *Dicranopteris* and provide an integrated framework to identify their ecological roles in the tropics and subtropics.

## Ecosystem Functions of *Dicranopteris*

### Colonization of the Habitat by *Dicranopteris*

**Figure 2:**
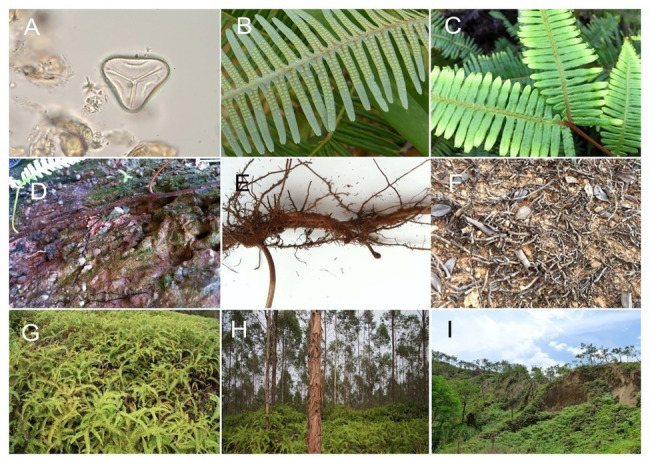
Individuals and communities of *Dicranopteris*. **(A)** Spores ×400. **(B)** Leaf blade and abaxial sporangia. **(C)** Leaf traits. **(D)** Underground stems (rhizomes). **(E)** Underground stems and roots. **(F)** Post-fire underground stems. **(G)** A *Dicranopteris* community on degraded land. **(H)** Understory *Dicranopteris* layer. **(I)** *Dicranopteris* cover in an area with soil erosion. Photographs were taken in Eucalyptus forests, Pinus forests, or degraded shrubland in subtropical China.

After a rainfall event, *Dicranopteris* can recolonize almost the entire surface area of a degraded and thus patchily colonizes a habitat. *Dicranopteris* species can reproduce *via* spores and clonal growth (Lin, 2004). The number of *D. dichotoma* spores in the top 0–5 cm of soil can be as high as 200,000 cm^−3^, and these spores can germinate in warm and rainy season in subtropical China. For *D. linearis*, sexual reproduction *via* the germination of dispersed spores also appears to be important during the earlier stage of colonization; the main evidence is that at least three-quarters of the genotypes are produced *via* outcrossing (Russell, 1996). Although spore propagation is critical in primary succession, clonal propagation is a more important means of vegetative growth and allows the plants to cover the entire surface of the landscape (Figure 2). The elements nitrogen (N), phosphorus (P), potassium (K), and aluminum (Al) accumulate in *Dicranopteris* rhizomes, which benefits from clonal propagation (Chen and Zhong, 1991). The strong vegetative growth ability of *Dicranopteris* species allows them to quickly cover degraded land and form a community of a single dominant species. As indicated by low genetic diversity, clonal propagation and growth are the primary means by which *D. linearis* covers degraded landscapes (Russell et al., 1999). *Dicranopteris* species also have a high colonization ability as a result of their pulsed growth. With sufficient precipitation and soil nutrients, *Dicranopteris* can grow quickly. However, the species grow slowly in dry season and in barren soils, which suggests that this pulsed growth probably improves resource-use efficiency (Lin, 2004). Moreover, the near modular growth of the fronds provided by the dormant bud could be helpful in this process.

### Resistance to Environmental Stress and Changes

#### Resistance to Environmental Stress

*Dicranopteris* species, in contrast to most plants, can endure strong solar radiation, high temperatures, acidity, infertile soil, and drought, which can also thrive in humid environments (Russell, 1996). The desiccation tolerance of *D. linearis* is attributed to its high antioxidant capacity (Kavitha and Murugan, 2016). In addition, the maximum temperatures are substantially lower in a *Dicranopteris* cover than in surrounding areas (Negishi et al., 2006). Because of their very high N- and P-use efficiencies, *Dicranopteris* species can occupy the surfaces of infertile soil (Russell, 1996). During primary succession, *Dicranopteris* communities alter the soil and microenvironment of the area. *Dicranopteris* species are more tolerant than other species to drought stress because of their higher water-use efficiency. In *Pinus* forests, the water-use efficiency of *Dicranopteris* reached 8 μmol·CO_2_·mmol^−1^ H_2_O, which was higher than that of most other understory species (Zhang et al., 2010).

#### Resistance to Soil Erosion Caused by Rainfall

*Dicranopteris* communities are beneficial to ecosystems by conserving water and soil in the following ways (Chen et al., 2016; Chen and Chen, 2018; Jiang et al., 2019). First, the leaves of a *Dicranopteris* can reduce splash erosion. Second, the rhizomes and adventitious roots are arranged in a crisscross pattern, which reduces linear erosion and sheet erosion from rainfall runoff. Third, the roots of *Dicranopteris* are short and highly branched and tend to hold soil particles and aggregates in place. In the absence of the aboveground parts of *Dicranopteris*, the nonflammable rhizomes and adventitious roots can firmly fix the surface soil following fire events. For instance, *D. linearis* (Zheng et al., 2008), *D. curranii* (Negishi et al., 2006), and *D. dichotoma* (Yue et al., 2014) reduced soil erosion by reducing runoff, mitigating splash and hydraulic surface erosion processes, and trapping sediment.

#### Resistance to Alien Species Invasion

Several growth and physiochemical traits are shared by *Dicranopteris* and invasive species, which include a preference for strong solar radiation, rapid growth, a strong reproductive ability, and allelopathic effects. As a consequence, *Dicranopteris* species can effectively compete with invasive species. More specifically, *Dicranopteris* species exhibit dense growth and always form a community with a single dominant species. The dense canopy and understory clonal stems occupy a large area and shade the microenvironment, which can limit the growth of other species. Because of their rapid clonal growth and spores reproduction, *Dicranopteris* species can also inhibit the establishment of alien species *via* nutrient limitation and the impediment of recruitment (Russell et al., 1999). In a Hawaiian rainforest, for example, alien species were unable to establish in *Dicranopteris*-colonized areas but were able to establish after *Dicranopteris* removal (Russell et al., 1998). *Dicranopteris* species also have strong allelopathic effects, which may inhibit the survival and growth of alien species (Zhang et al., 2004). In South China, for example, an aqueous extract of *Dicranopteris* was found to inhibit the germination and seedling growth of *Wedelia trilobata* and *Mikania micrantha*, two invasive species in this region (Zhao et al., 2007; Wu, 2008). Researchers have even suggested that *Dicranopteris* allelochemicals might be used to develop bioherbicides and potential insecticide to control alien species invasions (Zhao et al., 2007). Although the role of allelochemicals of *Dicranopteris* are considered important, their underlying mechanisms are still unclear. With regards to the presence of phytoecdysteroids, studies were carried out in some species of Gleicheniaceae (Hikino and Hikino, 1970; Russell and Fenemore, 1970). The major secondary compounds as allelochemical in bracken fern *Pteridium* included proanthocyanidin selligueain A (de Jesus Jatoba et al., 2016), phenolics, and condensed tannins (Alonso-Amelot et al., 2004).

#### Mitigation of Soil Contamination and Toxicity

Ferns are always considered as heavy metal accumulators, and some are found to be hyperaccumulators. For example, it was found that bracken fern (*Pteridium aquilinum*) can accumulate chromium (Cr) and nickel (Ni) in rhizome and fronds (Kubicka et al., 2015), and Chinese brake fern (*Pteris vittata*) is a hyperaccumulator of arsenic (Chen et al., 2002). *Dicranopteris* always grows in acidic soils with high contents of Al and manganese (Mg). Al and P combine to form aluminum phosphate, which is thought to reduce P availability. *Dicranopteris* can absorb Al, which reduces Al toxicity and increases P-use efficiency (Russell et al., 1998). *Dicranopteris* can also remove soil pollutants, such as heavy metal elements, rare earth elements, and radioactive elements. Many studies have shown that *Dicranopteris* has a strong ability to accumulate lead (Pb; Bi et al., 2006). The concentration of Pb in *Dicranopteris* was much higher in plants growing in lead-zinc mine wasteland (59.5 mg/kg) than in a non-polluted site (5 mg/kg; Bi et al., 2006) and even reached 556 mg/kg in the tungsten mine area of South China (Liu et al., 2008). *Dicranopteris* was also found to absorb zinc (Zn), tungsten (W), manganese (Mn), and cadmium (Cd) in the mining area of South China (Liu et al., 2008; Yang et al., 2012) and to assimilate rare earth elements, such as lanthanum (La), cerium (Ce), praseodymium (Pr), and neodymium (Nd; Wang et al., 203). *D. linearis* can even absorb radioactive elements, such as radium (Ra) and thorium (Th; Chao and Chuang, 2011; Song et al., 2014). *Dicranopteris* can be used to mitigate soil contamination and toxicity due to its high biomass accumulation, rapid growth, strong adaptability, and high bioconcentration ability (1.88–2.74; Song et al., 2014; Table 1).

**Table 1:** Soil contaminated types and elements accumulated by *Dicranopteris* species.

Soil contaminated types	Elements	*Dicranopteris* species	References
Al toxicity	Al	*Dicranopteris linearis*	Russell et al., 1998
Heavy metal	Zn	*Dicranopteris dichotoma*	Liu et al., 2008; Yang et al., 2012
	Cd	*D. dichotoma*	Liu et al., 2008; Yang et al., 2012
	Mo	*D. dichotoma*	Liu et al., 2008
	Cu	*D. dichotoma*	Liu et al., 2008
	Pb	*D. dichotoma*	Bi et al., 2006; Chen et al., 2011; Yang et al., 2012
	W	*D. dichotoma*	Liu et al., 2008
Semimetal	As	*D. dichotoma*	Wei et al., 2007
	Ba	*D. dichotoma*	Koyama et al., 1987
Rare earth	La	*D. dichotoma*	Wang et al., 2003
	Ce	*D. dichotoma*	Wang et al., 2003
	Pr	*D. dichotoma*	Wang et al., 2003
	Nd	*D. dichotoma*	Wang et al., 2003
Radioactivity	Ra	*D. linearis*	Chao et al., 2011; Song et al., 2014
	Th	*D. linearis*	Chao et al., 2011; Song et al., 2014

## Recognition of the Ecosystem Functions of *Dicranopteris*: Ecosystem Sustainability

### Carbon and Soil Nutrient Sequestration

*Dicranopteris* can survive for decades (Lin, 2004). The death of a large *Dicranopteris* clone provides a pulse of organic matter to the detrital pool and alters forest floor microenvironments, thus influencing species-specific colonization dynamics, organic matter, and nutrient cycling processes (Russell et al., 1999). In addition, *Dicranopteris* communities can reduce nutrient loss through runoff and leaching (Zheng et al., 2008). *Dicranopteris* communities have also been found to accelerate litter decomposition (Wu et al., 2011; Liu et al., 2012; Zhao et al., 2012; Yang et al., 2014). In open forests, forest litterfall can be intercepted by *Dicranopteris* communities before it reaches the ground; the litter decomposition rate was lower in the top part than in the bottom part of the communities, and the overall decomposition rate was faster in the communities than outside of the communities (Yang et al., 2014). Functioning as catalyzers, *Dicranopteris* communities increase carbon cycling and soil carbon accumulation in the whole ecosystem *via* aboveground-underground carbon flow (Guan, 2001; Wan et al., 2014). Soil organic carbon content was found to significantly increase in association with *Dicranopteris* communities, and dissolved organic carbon (DOC) and light fraction organic carbon (LFOC) were two and five times higher, respectively, in areas with than without *Dicranopteris* communities (Jiang, 2013). Soil organic carbon and total nitrogen content with *Dicranopteris* (49.01 and 2.37 t ha^−1^) was obviously higher than that without *Dicranopteris* (28.11 and 1.65 t ha^−1^) in 30-year *Pinus massoniana* forest (Jiang, 2013). It was also showed that soil nitrate increased after bracken fern *P. aquilinum* invasion (DeLuca et al., 2013; Bardon et al., 2018). In addition, decreases in the litter decomposition rates led to reductions in nutrient availability in a series of *Dicranopteris* removal experiments (Negishi et al., 2006; Zhao et al., 2012). These results indicate that *Dicranopteris* communities increase carbon and soil nutrient sequestration.

### Causes of Regeneration and the Control of Ecological Thresholds

**Figure 3:**
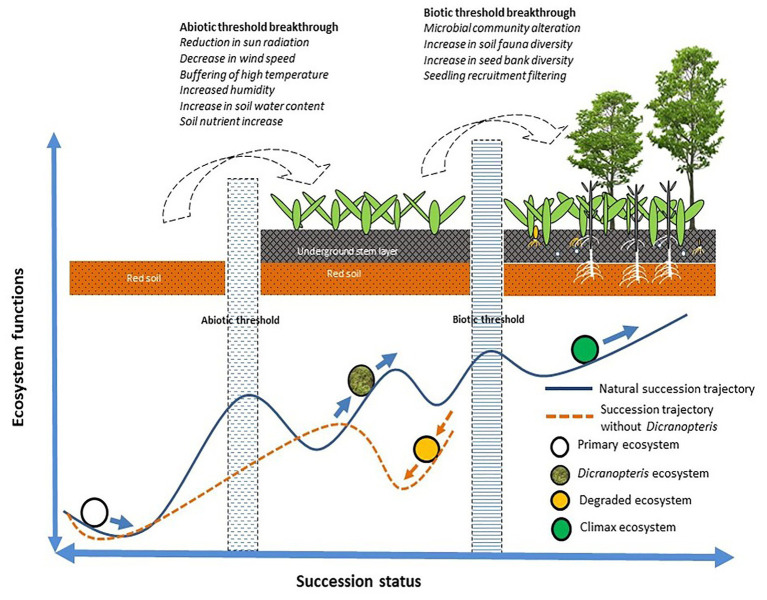
A model of how *Dicranopteris* communities facilitate to overcome the abiotic and biotic thresholds of succession (based on Sun et al., 2013).

For succession to proceed or for restoration to be successful, certain abiotic and biotic thresholds must be surpassed (Ren et al., 2006). As indicated in Figure 3, *Dicranopteris* species enable succession to proceed *via* abiotic effects (by reducing solar radiation, wind speed, and high temperatures, and by increasing humidity and soil moisture and nutrient content) and *via* biotic effects (by altering the soil microbial and faunal community, by increasing soil seed bank diversity, and by the screening of seedlings; Ren et al., 2006). *Dicranopteris* was considered as an ecological filter (Zhang et al., 2010). *Dicranopteris* species improve soil structure and increase soil nutrient content by reducing water and soil loss and by fixing carbon and nitrogen (Guan, 2001; Xie et al., 2006; Chen et al., 2014). *Dicranopteris* species increase the diversity in the soil seed bank by intercepting the “seed rain” compared with bare ground. This “root sieve” is more than 10 cm deep and insulates the aboveground part of *Dicranopteris* and the soil layer (Zhang et al., 2010). The seeds retained by the aboveground part are intercepted by the underground part. These seeds eventually settle through various layers, which represent a physical, chemical, and biological screening. Seeds that pass through these screens may germinate, and the resulting seedlings may infiltrate the *Dicranopteris* communities. These species can shape the soil seed and seedling bank at the later stages of ecological succession. Most species cannot recruit in *Dicranopteris* communities due to the allelopathic effects of *Dicranopteris* species (Ismail and Chong, 2007). *Dicranopteris* may have allelopathic effect on two invasive species, *Bidens pilosa* and *Eupatorium catarium* in South China, and the allelochemical, such as 2,3-Butanediol and 1,2,3-propanetriyl ester, which may play an important role in inhibitory effects of invasive species (Juma et al., 2019). This phenomenon of ecological filter was found in other ferns. Bracken fern *Pteridium* were also regarded as ecological filters worldwide (Senyanzobe et al., 2020). The seedling establishment of rainforest species in Brazil, Scots pine and Norway spruce in Sweden, and tropical species in Africa can be significantly interfered by *Pteridium* (Dolling, 1996; Silva Matos and Belinato, 2010; Ssali et al., 2019). Proanthocyanidin selligueain A is the major secondary compound in the green fronds and litter of *Pteridium* (de Jesus Jatoba et al., 2016).

### Successional Facilitation

Successional trajectories are determined by stable environmental factors, such as air temperature, precipitation, geological conditions, and soil type, and even by the presence of stable keystone species. Under global climate change, these stable factors may change to some degree, thereby altering interspecific associations and successional trajectories. On sunny slopes in South China, *D. dichotoma* biomass can reach 11,320 kg/ha (Yue, 2009). As previously noted, *Dicranopteris* grows best in environments with high light penetration, such as on bare land or in open forests, and *Dicranopteris* thickets are known to persist for decades once established (Russell, 1996). Shade intolerance is the Achilles’ Heel of *Dicranopteris*. *Dicranopteris* species can protect the successional process by relinquishing their dominance as the canopies of the dominant species in later successional stages increasingly reduce light penetration. The successional trajectory always progresses from ferns to shrubs and then to broad-leaved forests in the tropics and subtropics. *Dicranopteris* species are always dominant during the earlier-successional stage. An interesting study in subtropical China showed that the shrub *Rhodomyrtus tomentosa* was the first plant to colonize a burned, degraded area (Yang et al., 2010); when all of the shrubs were killed by a parasitic vine (*Cassytha filiformis*), the bare area was invaded and covered by *Dicranopteris*. Ban et al. (2008) concluded that the entire successional process began with the presence of *Dicranopteris*.

## Future Research Directions Regarding the Ecosystem Functions of *Dicranopteris*

### Long-Term and Global-Scale Research Framework/Agenda

**Figure 4:**
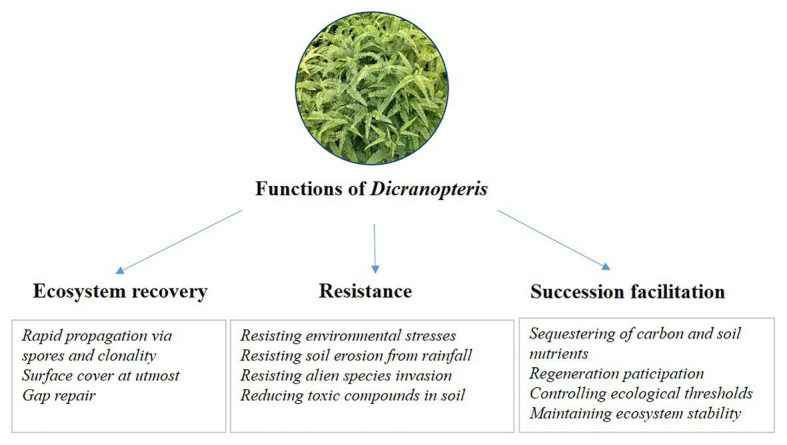
The proposed ecological functions of *Dicranopteris*, including ecosystem recovery, resistance, and succession facilitation.

Long-term research can increase our understanding of changes in ecosystem structure and function (Kuebbing et al., 2018). To date, long-term studies of *Dicranopteris* are rare. Although studies lasting 1–2 years have been conducted with *Dicranopteris* (Wu et al., 2011; Zhao et al., 2012; Yang et al., 2014), such short-term investigations, especially when involving *Dicranopteris* removal, cannot fully explain the ecosystem functions of these species. For example, successional trajectory protection may be the key ecosystem function of *Dicranopteris*, but this protection becomes manifested after more than 1 or 2 years. Another example considers the role of *Dicranopteris* in ecological filtering. This key ecosystem function is a long-term ecological process involving the effects of communities on seed retention, seed germination, seedling screening, and seedling establishment. Although published reports support the model in Figure 3 and the ecological functions listed in Figure 4, long-term research is greatly needed to clarify the ecological functions of *Dicranopteris* species and their effects on forest succession in tropical and subtropical regions.

*Dicranopteris* can be considered as a global pioneer species in the tropics and subtropics. However, almost all studies on *Dicranopteris* have been conducted at the local scale, and global-scale research on *Dicranopteris* is still lacking. A global-scale research network for the genus would be useful. Current global-scale research networks concerning various ecological and evolutionary topics include the International Long Term Ecological Research Network (ILTER), the Global Biodiversity Information Facility (GBIF), the Center for Tropical Forest Science - Forest Global Earth Observatory (CTFS-Forest GEO), and the International Canopy Network (ICN). A global research network would be useful for answering a variety of fundamental questions. For example, Why are the number of *Dicranopteris* species so small? Is that attributed to the low genetic diversity in all *Dicranopteris* species? Will the distribution of *Dicranopteris* expand under the scenario of changing global environments?

### Fusion of Ancient and Modern Data

Information on species co-occurring with *Dicranopteris* spores can, therefore, be used as an indicator of how the ecological role of the genus may have changed under different climates. For example, the combination *Dicranopteris-Cibotium-Castanopsis* in the stratigraphic record indicates a humid environment with tropical and subtropical ferns, while the combination *Pinus-Castanopsis-Quercus-Gramineae-Dicranopteris* indicates tropical and subtropical grasslands (Peng et al., 2015). The current presence of *Dicranopteris*, in contrast, indicates early stages of succession in tropical and subtropical forests. *Dicranopteris* spores are consistently found in various strata, suggesting the important role of these species in the past (Sun et al., 2016). Big data regarding *Dicranopteris* from worldwide strata should be reanalyzed, and ancient and modern data should be combined to determine how the ecological roles of *Dicranopteris* species may change in the future.

### Predicted Expansion and Functions of *Dicranopteris* in a Changing Environment

**Figure 5:**
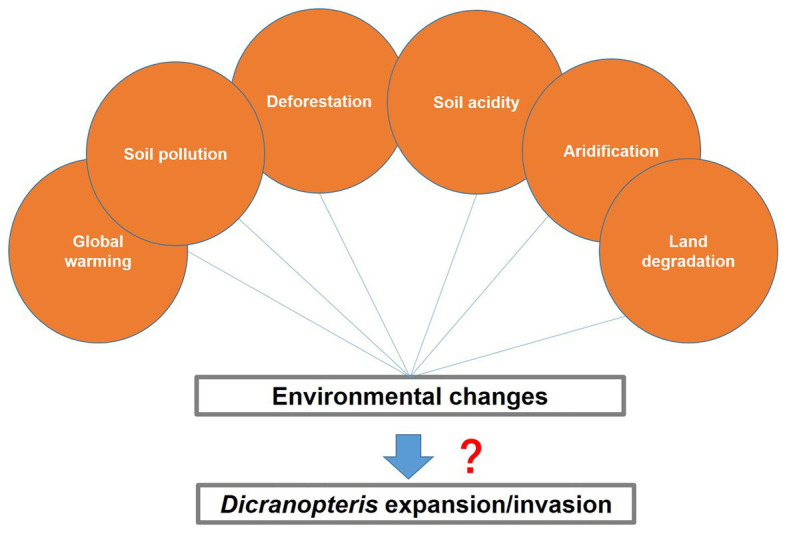
Environmental changes that may lead to *Dicranopteris* expansion or invasion.

The Earth’s environment is changing *via* global warming, land degradation, deforestation, aridification, nitrogen deposition, soil acidification, soil pollution, etc. Further research is needed to answer the following questions relevant to global change: Can stable species, such as *Dicranopteris*, act as refuges that facilitate the establishment of unstable species under the scenario of global change? What are the mechanisms of *Dicranopteris* expansion? Can *Dicranopteris* adapt to the changing global environments? Few studies referred to the impact of global change to *Dicranopteris* in the past. Another bracken fern *Pteridium* species (such as *Pteridium aquilinum*) that is similar with *Dicranopteris* have been regarded as aggressive and invasive pioneer plant species in a changing world (Gaudio et al., 2011). Although warming and elevated CO_2_ did not change the growth of *Pteridium* (Whitehead et al., 1997; Caporn et al., 1999), nitrogen deposition and drought frequency may affect it (Gordon et al., 1999). Temperatures for *Dicranopteris* are generally more suitable in tropical than in temperate regions. With global warming, the climatic zone of the tropics is expected to move to high-latitude areas, which may cause *Dicranopteris* to evolve temperature adaptability. Additionally, deforestation and land degradation frequently occur in the tropics and subtropics, and thereby increase exposures to sunlight and the production of xeric environments. These conditions are consistent with the sun-loving and drought-tolerant traits of *Dicranopteris*. The establishment of most plant species except for *Dicranopteris* species is prevented in soils that have experienced high levels of nitrogen deposition, acid deposition, and heavy metal contamination. Therefore, the expansion of *Dicranopteris*, similar to that of bamboo, can be predicted to occur in the changing environments of the future. Future research is needed to test these hypotheses and to fill the gaps (Figure 5).

## Author Contributions

LY, YH, ZS, and HR conceived the study. LY, LL, ML, ZS, YH, JW, and NL analyzed the data and contributed reagents, materials, and analysis tools. LY, YH, LL, ZS, JW, NL, and HR wrote the paper. All authors contributed to the article and approved the submitted version.

### Conflict of Interest

The authors declare that the research was conducted in the absence of any commercial or financial relationships that could be construed as a potential conflict of interest.
