# Corrigendum: Rethinking the Ecosystem Functions of *Dicranopteris*, a Widespread Genus of Ferns

**DOI:** 10.3389/fpls.2021.770556

**Published:** 2021-10-05

**Authors:** Long Yang, Yuhui Huang, Lucas Vieira Lima, Zhongyu Sun, Meijie Liu, Jun Wang, Nan Liu, Hai Ren

**Affiliations:** ^1^Guangdong Open Laboratory of Geospatial Information Technology and Application, Guangdong Academy of Sciences, Guangzhou Institute of Geography, Guangzhou, China; ^2^Guangdong Provincial Key Laboratory of Forest Silviculture, Protection and Utilization, Guangdong Academy of Forestry, Guangzhou, China; ^3^Departamento de Botânica, Laboratório de Sistemática Vegetal, Instituto de Ciências Biológicas, Universidade Federal de Minas Gerais, Belo Horizonte, Brazil; ^4^Key Laboratory of Vegetation Restoration and Management of Degraded Ecosystems, South China Botanical Garden, Chinese Academy of Sciences, Guangzhou, China

**Keywords:** *Dicranopteris*, ecosystem function, facilitation and competition, ecosystem resilience, succession facilitation, tropical forest

In the original article, there was a mistake in Figure 1, Global distribution of *Dicranopteris* species as published with national boundary problems. The corrected Figure 1 Global distribution of *Dicranopteris* species appears below.

**Figure 1:**
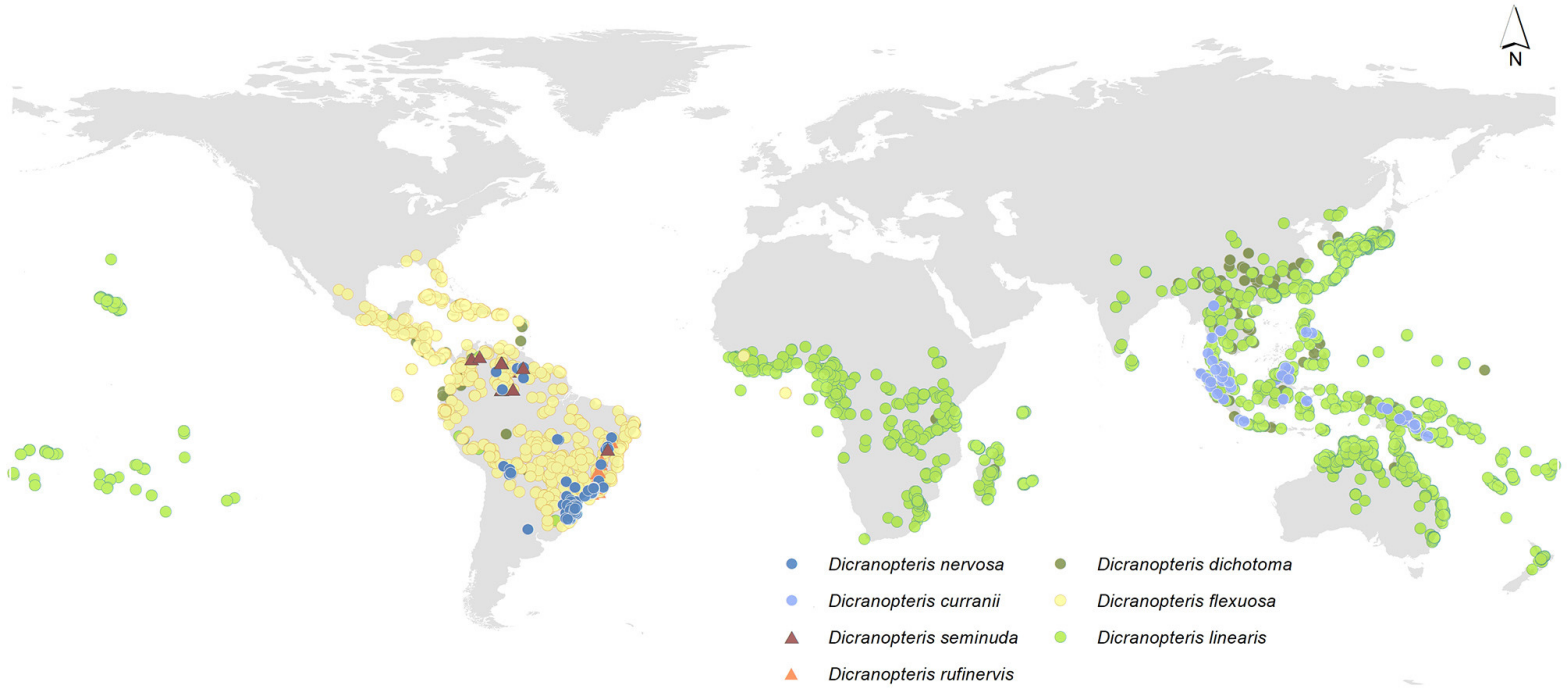
Global distribution of *Dicranopteris* species, including *Dicranopteris dichotoma* (deep green dots), *Dicranopteris curranii* (shallow blue dots), *Dicranopteris flexuosa* (yellow dots), *Dicranopteris seminuda* (brown triangles), *Dicranopteris nervosa* (navy blue dots), *Dicranopteris rufiervis* (orange triangles), and *Dicranopteris linearis* (green dots). The distribution information was mainly obtained from Global Biodiversity Information Facility (gbif.org), sampling by Long Yang in East Asia and Lucas Vieira Lima in the neotropics.

The authors apologize for this error and state that this does not change the scientific conclusions of the article in any way. The original article has been updated.

## Publisher's Note

All claims expressed in this article are solely those of the authors and do not necessarily represent those of their affiliated organizations, or those of the publisher, the editors and the reviewers. Any product that may be evaluated in this article, or claim that may be made by its manufacturer, is not guaranteed or endorsed by the publisher.

